# The impact of a digital self-care intervention based on mHealth on reducing sleep disorders during pregnancy: a quasi-experimental controlled study

**DOI:** 10.1186/s12978-025-02238-0

**Published:** 2025-12-24

**Authors:** Mahdieh Naderi, Zahra Alipour, Zohre Khalajinia, Zohre Momenimovahed

**Affiliations:** 1 Department of Midwifery, Student Research Center, Qom University of Medical Sciences, Qom, Iran; 2 Assistant Professor of Reproductive Health, Spiritual Health Research Center, Qom University of Medical Sciences, Qom, Iran; 3 Associate Professor of Reproductive Health, Spiritual Health Research Center, Qom University of Medical Sciences, Qom, Iran; 4 Professor of Reproductive Health, Spiritual Health Research Center, Qom University of Medical Sciences, Qom, Iran

**Keywords:** Pregnancy, Sleep disorders, Self Care, mHealth, Iranian Traditional Medicine, Health literacy

## Abstract

**Background:**

Sleep disorders affect up to 80% of pregnant women, leading to risks such as preeclampsia and preterm birth, especially in low- and middle-income countries such as Iran. Mobile health (mHealth) digital self-care interventions can provide accessible solutions; however, few incorporate traditional medicine. This study assessed the effectiveness of a digital self-care intervention utilizing mobile health (mHealth) to enhance sleep quality and improve health literacy in pregnant women.

**Methods:**

This quasi-experimental controlled trial was conducted from April to August 2025 in Qom, Iran, and involved 72 pregnant women from a community health center. Participants were randomly assigned to either the intervention (*n* = 36) or control (*n* = 36) groups.

The intervention included twice-weekly digital sessions via messaging apps (Eita and Telegram), focusing on six areas of Iranian traditional medicine for pregnancy: nutrition, mental health, physical activity, sleep, environmental factors, and bodily substance retention. The sessions featured audio files, videos, pamphlets, reminders, and question-and-answer sessions over three months.

Sleep quality was measured using the Pittsburgh Sleep Quality Index (PSQI), and health literacy was assessed using the Maternal Health Literacy Questionnaire at baseline and three months post-intervention. Data analysis was performed using SPSS version 26 with t-tests and chi-square tests (p<0.05).

**Results:**

Baseline demographics and outcomes were comparable between the groups (p>0.05). Post-intervention, the intervention group's global PSQI score improved significantly from 7.22±3.29 to 4.41±2.75 (p < 0.001), indicating better sleep quality, whereas the control group’s score worsened from 6.58±3.02 to 7.11±3.45 (p=0.32). Improvements were observed in most PSQI subscales in the intervention group. Maternal health literacy increased from 59.94±8.23 to 68.16±4.76 in the intervention group (p < 0.001) compared to a minor increase from 61.38±5.89 to 63.89±4.69 in controls (p=0.02). The between-group differences were significant for all improved outcomes (p<0.05).

**Conclusions:**

This culturally tailored mHealth intervention can improve sleep quality and health literacy in pregnant women by integrating traditional Iranian practices with digital tools. It offers an affordable model for pregnant women to enhance their maternal well-being.

## Introduction

Research on digital self-care educational programs utilizing mHealth technology to enhance maternal health during pregnancy has emerged as a significant area of investigation owing to its potential for improving access to pregnancy care outcomes [1, 2]. The integration of mobile health applications into maternal health care has evolved considerably in recent years, with early systems primarily focusing on reminders and education for prenatal care [3, 4]. Recent studies emphasize the importance of interactive and culturally tailored interventions that address the physical and psychological aspects of pregnancy [5, 6]. This evolution highlights the growing recognition of the role of digital health in reducing maternal morbidity and mortality, particularly in low- and middle-income countries (LMICs), where traditional access to healthcare remains limited [7].

Sleep disorders are one of the most prevalent issues during pregnancy, with rates rising from 63% in the first trimester to 80% in the third trimester [8]. In developing countries such as Iran, the prevalence of sleep disorders among pregnant women is 61.9% [9]. This condition is linked to adverse maternal and perinatal outcomes, including depression, cesarean delivery, preeclampsia, gestational diabetes, preterm birth, low birth weight, offspring obesity, and hypertension [10, 11]. Given the extensive penetration of smartphones in LMICs, digital self-care programs offer a promising avenue for enhancing maternal well-being [7].

Maternal health literacy (MHL), defined as the ability to access, understand, appraise, and apply health information during pregnancy, plays a pivotal role in promoting positive maternal outcomes [12, 13]. Low MHL is associated with poor adherence to prenatal care, increased stress, and a heightened risk of mental health issues, such as antenatal depression and anxiety, which can exacerbate sleep disturbances [13, 14]. Studies have indicated that pregnant women with higher health literacy are more likely to engage in health-promoting behaviors, including better sleep hygiene practices, leading to improved sleep quality and overall psychological well-being [15]. For instance, interventions that enhance MHL have been shown to reduce the risk of antenatal depression through better stress and sleep self-management [16]. In LMICs such as Iran, where cultural and socioeconomic barriers limit health education, elevating MHL through accessible tools can mitigate these effects, fostering resilience against sleep disorders and mental health challenges [17, 18]. By empowering women to recognize symptoms early and adopt evidence-based strategies, improved MHL not only enhances sleep duration and efficiency but also bolsters emotional regulation, thereby reducing the incidence of postpartum mental health complications [13, 18].

Despite the expansion of pregnancy-related mobile health applications, substantial challenges persist in ensuring their quality, cultural relevance and efficacy. Studies have revealed that many existing applications lack comprehensive content, professional input, and evidence-based validation [19, 20]. Moreover, although mobile health interventions have demonstrated benefits in promoting self-care behaviors and adherence to antenatal care [21], gaps remain in addressing sleep quality disorders and incorporating the principles of Traditional Iranian Medicine into digital platforms to ameliorate these issues [2, 22]. Debates also exist regarding the balance between technological innovation and the preservation of traditional healthcare knowledge [23]. Consequently, there is a vital need to evaluate digital self-care educational programs that integrate frameworks from Traditional Iranian Medicine to enhance sleep quality and maternal health during pregnancy.

The incorporation of these concepts into mobile health platforms seeks to improve sleep quality and overall maternal well-being by blending evidence-based digital education with culturally aligned traditional practices [23]. This framework informs the assessment of program effectiveness and user engagement. Accordingly, the present study was undertaken to evaluate the effectiveness of a digital self-care intervention utilizing mobile health (mHealth) to enhance sleep quality and improve health literacy in pregnant women.

## Methods

### Study design and population

This quasi-experimental controlled study aimed to evaluate the effectiveness of a digital self-care intervention utilizing mobile health (mHealth) to enhance sleep quality and improve health literacy in pregnant women. The study was approved by the Ethics Committee of Qom University of Medical Sciences (IR.MUQ.REC.1403.292) and involved 72 pregnant women who received prenatal care in Qom, Iran. The study was conducted from April to August 2025.

The inclusion criteria for participants were as follows: access to a mobile phone with internet connectivity, sufficient literacy (at least an elementary level), singleton pregnancy, being married and satisfied with marital life, maintaining a normal body mass index, having no known psychiatric disorders or use of psychiatric medications, not experiencing sleep disorders, and not using sleep-inducing drugs, narcotics, alcohol, or psychedelics. Additionally, participants were not to be employed in rotating or night shifts, must not have high-risk pregnancy criteria, had no history of infertility or miscarriage, and their spouses had no physical or mental illnesses.

Exclusion criteria included failure to complete educational sessions, failure to respond to post-test questionnaires, or experiencing pregnancy complications that required immediate medical attention.

The sample size was calculated using the formula for comparing two independent means in quasi-experimental studies: $\:n=\frac{\left[Z\right(1-\alpha\:/2)+Z(1-\beta\:\left){]}^{2}\right(S{D}_{1}^{2}+S{D}_{2}^{2})}{{d}^{2}}\:\:$where α = 0.05 (Z(1-α/2)=1.96), power=80% (1-β = 0.8, Z(1-β)=0.84), SD1=4.45, SD2=4.77, and minimum detectable difference d = 3.5 (based on mean sleep quality scores from Rohani et al., 2019) [24], yielding *n* = 27 per group. Accounting for 10% attrition, the final sample was increased to 36 participants per group (total *N* = 72).

Samples were selected from clients attending community health centers in Qom after obtaining written, informed consent. Multistage cluster sampling was conducted. First, two main Health Centers (Health Centers 1 and 2) were considered clusters. From the 14 comprehensive health centers under Health Center 1, five high-traffic centers (based on the number of clients) were randomly selected, and similarly, five were selected from the 14 centers under Health Center 2. The researcher regularly visited these centers on weekdays (excluding holidays) and randomly selected eligible pregnant women attending prenatal care using convenience sampling, which may have introduced a risk of selection bias. Subsequently, the participants were randomly assigned to the intervention and control groups (36 each). For blinding, prenatal care providers were unaware of the participants’ group assignments, and the data analyst operated in a blinded manner. Before implementing the educational intervention program, the research units’ sleep quality and pregnancy health literacy were assessed and reassessed three months after the digital self-care education based on Iranian traditional medicine teachings.

### Intervention

The educational approach in this intervention involved implementing digital self-care sessions based on Iranian traditional medicine teachings using a mobile health (mHealth) platform [25]. The educational program comprises six assertive sessions, held three times a week over two weeks. Each session features thematic sections, including pre-tests and post-tests across six key domains: food and drink, mental and emotional health, movement and rest, sleep and wakefulness, air and environment, and substance retention and elimination, all focused on pregnancy-related changes. First Session - Objectives: Participants will complete a questionnaire, introduce themselves, and become acquainted with the program’s content and objectives, emphasizing the role of traditional medicine and exercise guidelines during pregnancy. Topics Covered: Overview of research interventions, the vital importance of exercise in pregnancy, and practical timing recommendations. Teaching Method: Individual delivery of the questionnaire and pre-test via messaging apps (Eita or Telegram), alongside educational pamphlets and videos on movement and rest, followed by the post-test after each session. Second Session- Objectives: Understand the importance of healthy air for pregnancy health and how to manage various weather conditions (cold, hot, and polluted air). Develop skills in therapeutic breathing. Topics Covered: The benefits of healthy air; preventing complications from heat exposure and frostbite; addressing air pollution; and maintaining clean indoor air. Teaching Method: Individual pre-tests, educational files on air and environment care sent via Eita or Telegram, a video on therapeutic breathing, and individual post-tests. Third Session - Objectives: Familiarization with psychological states during pregnancy and their impact on health, emotional balance, and mental well-being. Topics: Emotional Balance and Strengthening Mental Health. Teaching Method: Individual pre-tests; provide educational content on psychological care via Eita or Telegram; share an audio file on mental health; individual post-tests. Fourth Session - Objectives: To understand the significance of proper nutrition and hydration during pregnancy, including what to eat, what to avoid, and proper eating etiquette. Topics covered were the impact of diet on fertility, recommendations based on digestion and food interactions, and appropriate behaviors while eating and drinking. Teaching Method: Distributing a pre-test to members, sharing content on nutrition during pregnancy via educational pamphlets and audio in the Eita or Telegram channel, and sending a post-test to members. Fifth Session - Objectives: Understanding body cleansing and waste excretion during pregnancy, including bowel movements, urination, sweating, and bathing. Topics: Natural cleansing and effective ways to eliminate unnecessary materials. Teaching Method: Pre-test distribution to members, educational content shared via Eita or Telegram, an audio file on natural cleansing sent to the channels, and post-test distribution to members. Sixth Session – Objectives: Understand proper sleep conditions during pregnancy and their health effects. Topics covered include sleep patterns, duration, timing, nutrition, pre-sleep routines, sleeping environments, and covering during sleep.

Teaching Method: Distributing a pre-test to each member, providing educational pamphlets on sleep during pregnancy via the Eita or Telegram channel, sharing an audio file on proper sleep conditions, and sending a post-test individually. To reinforce education and promote a healthy lifestyle, three months were designated in accordance with the recommendations of the content compilers [25]. The intervention group received educational content in the form of audio files, instructional videos, and related pamphlets via accessible messaging applications such as Eita and Telegram. Additionally, during the three-month follow-up period, the researcher sent reminder SMS messages on session topics to the intervention group. Question-and-answer programs regarding the educational content were also arranged on Telegram and Eita for women in the intervention group.

The control group received only routine prenatal care education, which included standard guidance on nutrition, physical activity, prenatal check-ups, and general health monitoring as provided by community health centers in Iran, and no additional interventions were performed. To adhere to ethical considerations for the control group, two general educational sessions on prenatal care were held. The schedule of educational sessions, including recommendations from Iranian traditional medicine for maintaining and promoting health during pregnancy, is presented in Table 1.

**Table 1:** Educational sessions program

Session	Objectives	Topics	Teaching Method
First Session	-Filling out the questionnaire -Self-introduction to members -Familiarizing members with the contents of the educational intervention, educational objectives, and how to conduct pre-test and post-test. -Familiarizing with the growing position of traditional medicine in the health care system of Iran and the world -Familiarizing with proper exercise and exercise conditions at different times of pregnancy	-Introduction: General explanation about research interventions - Position of traditional medicine in Iran and the world -Importance of exercise during pregnancy - Best time to exercise - Recommendations before exercise - Recommendations during exercise - Recommendations after exercise	-Sending the questionnaire through available messaging programs, such as Eita or Telegram, to each member individually -Sending the pre-test related to the session content to each member individually. Preparing content related to care in terms of movement and rest during pregnancy in the form of an educational pamphlet and sending it to the Eita or Telegram channel -Sending an educational video regarding how to perform exercises during pregnancy to the Eita or Telegram channel. - Sending the post-test related to the session content to each member individually
Second Session	-Understanding the importance of healthy air in maintaining health during pregnancy and measures for dealing with disrupted weather conditions, including cold, hot, and polluted air. - Acquiring skills in performing therapeutic breathing during pregnancy	-Role of healthy air -Moderate air -Hot air - Prevention of complications of hot air and heatstroke in pregnancy -Cold air - Prevention of complications of cold air and frostbite in pregnancy -Air pollution: Measures for living in areas with polluted air - Keeping the home air clean	-Sending the pre-test related to the session content to each member individually - Preparing content related to care in terms of air and environment in the form of an educational file and sending it to the Eita or Telegram channel - Sending an educational video regarding how to perform therapeutic breathing during pregnancy to the Eita or Telegram channel - Sending the post-test related to the session content to each member individually
Third Session	-Familiarizing with various psychological states during pregnancy and ways to control them, and their important role in maintaining health and the occurrence of diseases - Balance in emotional state during pregnancy -Strengthening spiritual and mental health	-Balance in Emotional State During Pregnancy -Strengthening Spiritual and Mental Health	-Sending the pre-test related to the session content to each member individually -Preparing content related to care in terms of psychological and mental aspects during pregnancy in the form of an educational file and sending it to the Eita or Telegram channel -Sending an audio file regarding strengthening mental health during pregnancy to the Eita or Telegram channel - Sending the post-test related to the session content to each member individually
Fourth Session	-Familiarizing with the importance of eating and drinking as the most controllable measures for maintaining health and proper nutritional etiquette, and suitable foods during pregnancy	-Importance of eating and drinking during pregnancy -Foods harmful to fertility -Foods beneficial for fertility - Recommendations based on the type of digestion -Recommendations based on nutritiousness -Recommendations based on food interactions during pregnancy - Proper etiquette for eating food -Proper etiquette for drinking	-Sending the pre-test related to the session content to each member individually -Preparing content related to care in terms of eating and drinking during pregnancy and sending it in the form of an educational pamphlet to the Eita or Telegram channel - Sending an audio file regarding beneficial and harmful foods during pregnancy to the Eita or Telegram channel -Sending the post-test related to the session content to each member individually
Fifth Session	-Familiarizing with the importance of body cleansing and ways to excrete waste materials, including bowel movement, urination, sweating, bathing, etc., during pregnancy	-Natural cleansing Ways to excrete unnecessary materials during pregnancy, Bathing	-Sending the pre-test related to the session content to each member individually -Preparing content related to care in terms of excreting unnecessary materials in the form of an educational file in the Eita or Telegram channel -Sending an audio file regarding natural cleansing during pregnancy to the Eita or Telegram channel -Sending the post-test related to the session content to each member individually
Sixth Session	-Familiarizing oneself with proper sleep conditions during pregnancy and the effects of sleep on health, with attention to pregnancy	-Sleep and wakefulness -Good sleep. - How much to sleep? -When to sleep? -Sleep and food -What to do before sleep? -Where to sleep? -Covering during sleep	-Sending the pre-test related to the session content to each member individually -Preparing content related to care of sleep and wakefulness during pregnancy in the form of an educational pamphlet and sending it to the Eita or Telegram channel -Sending an audio file regarding proper sleep conditions during pregnancy to the Eita or Telegram channel -Sending the post-test related to the session content to each member individually

### Tools

Data were collected using the following instruments.

### Demographic and obstetric data collection checklist

The variables included maternal age, education level, employment status, economic status, gestational age, body mass index (BMI), and standard questionnaires for Pittsburgh sleep quality and pregnancy health literacy.

### Pittsburgh sleep quality index

Pittsburgh Sleep Quality Index (PSQI) Sleep quality was assessed using the Pittsburgh Sleep Quality Index (PSQI), an internationally standardized and validated self-rated questionnaire that measures sleep quality and disturbances over the preceding four-week period [26]. The PSQI comprises seven component scores (range 0–3 each), yielding a global score ranging from 0 to 21, with higher scores indicating poorer sleep quality. A global score of ≥ 6 is conventionally interpreted as poor sleep quality. The Persian version was previously validated in Iran, with established content validity and a reported Cronbach’s alpha of 0.77 [27].

Questionnaires were distributed online via messaging applications such as Eita and Telegram before the intervention began and three months after the sessions ended for both groups.

### Maternal health literacy questionnaire

Maternal health literacy was measured using the validated Persian adaptation of the Maternal Health Literacy Questionnaire originally developed by [28]. The Persian version, standardized by Kharrazi et al. for Iranian pregnant women, consists of 14 items scored on a 5-point Likert scale (1 = strongly disagree to 5 = strongly agree), producing a total score ranging from 14 to 70, with higher scores indicating greater health literacy [29].

The original instrument demonstrated a Cronbach’s alpha of 0.81 [28], whereas the Persian adaptation reported Cronbach’s alphas of 0.89 (maternal health literacy domain) and 0.67 (pregnancy domain) in the standardization study [29].

Questionnaires were administered online via Eitaa and Telegram messaging applications at baseline and three months post-intervention to both the intervention and control groups.

### Statistical analysis

Data were analyzed using SPSS software (version 26). Quantitative data were summarized using means and standard deviations, while nominal and ordinal data were summarized using frequencies and percentages. Data normality was assessed using the Kolmogorov-Smirnov test. Parametric tests (such as independent t-tests) were used to compare quantitative variables between groups. Chi-square tests were used for nominal and ordinal data analyses. Within-group comparisons of the mean scores for the dependent variables (pre- and post-intervention) were performed using paired t-tests. The significance level was set at less than 0.05 for all tests.

## Results

### Baseline characteristics

Based on the findings in Table 2, a comparative analysis of the baseline demographic and clinical characteristics between the intervention and control groups revealed no statistically significant differences across all variables examined (*p* > 0.05). These findings are detailed as follows: The mean age of participating women in the intervention group was 30.36 ± 6.8 years, compared to 32.16 ± 5.9 years in the control group, with no statistically significant difference (t = 0.4, *p* > 0.05). Similarly, the mean age of spouses in the intervention group was 36.30 ± 7.1 years, versus 36.9 ± 6.3 years in the control group, and this difference was also not significant (t = 1.1, *p* > 0.05). Additional comparative analyses of the baseline demographic and clinical characteristics of the intervention and control groups are presented in Table 2. The findings in Table 2 indicate that the two groups were well balanced and exhibited similar distributions across all baseline demographic and clinical characteristics.

**Table 2:** Comparison of baseline characteristics in study groups

	Intervention Group (*n* = 36)	Control Group (*n* = 36)	Statistical Test Results	Sig
	Mean (SD) or Number (%)	Mean (SD) or Number (%)		
Age of husband	36.30(7.1)	36.9(6.3)	t-test: 1.1	n.s.
Age of women	30.36(6.8)	32.16(5.9)	t-test: 0.4	n.s.
Gestational age	18.91(1.3)	18.67(1.3)	t-test: 0.76	n.s.
Body mass index	26.92(3.5)	28.19(4.9)	t-test: 1.24	n.s.
Education level of husband (%)			χ^2^: 0.41	n.s.
High school or less	17(47.2)	15(61.1)		
More than high school	19(52.8)	14(38.9)		
Education level of women (%)			χ^2^: 0.19	n.s.
High school or less	21(58.3)	29(80.6)		
More than high school	15(41.7)	7(19.4)		
Occupational status of women (%)			χ^2^: 0.9	n.s.
Employed	5(13.9)	4(11.1)		
Housewife	31(86.1)	32(88.9)		
Economic level (%)			χ^2^: 0.39	n.s.
Low	4(11.1)	8(22.2)		
Medium	26(72.2)	25(69.5)		
High	6(16.7)	3(8.3)		
Parity (%)			χ^2^: 0.56	n.s.
Primparity	8(22.2)	11(30.6)		
Multiparty	28(77.8)	25(69.4)		

### Maternal health literacy

A comparison of the mean maternal health literacy scores between the intervention and control groups at baseline and three months post-study revealed no significant difference at baseline (59.94 versus 61.38, respectively; *p* > 0.05), indicating homogeneity between the groups prior to the intervention. However, after three months of implementing the intervention, the mean health literacy score in the intervention group significantly increased (68.16 ± 4.76), whereas the increase in the control group was negligible (63.89 ± 4.69). An independent t-test confirmed that this difference was statistically significant (*p* < 0.001), demonstrating the positive impact of the digital self-care education intervention for a healthy lifestyle based on Iranian traditional medicine teachings on improving health literacy among pregnant women (Table 3).

**Table 3:** Comparison of maternal health literacy scores between the two groups at two times

	Intervention Group (*n* = 36)	Control Group (*n* = 36)	Statistical Test Results	Sig
	Mean (SD)	Mean (SD)		
Maternal Health Literacy Questionnaire				
At Intake	59.94(8.23)	61.38(6.12)	t-test: 0.84	n’s.
After 3 months	68.16(4.76)	63.89(4.69)	t-test: 3.84	0.000

### Sleep quality

Regarding sleep quality, a comparison of scores across the various subscales of the Pittsburgh Sleep Quality Index (PSQI) at baseline and three months post-intervention showed no significant differences between the groups in any subscale at baseline (*p* > 0.05). However, after three months, all subscales of sleep quality, except for the use of sleep medication, exhibited significant improvements in the intervention group compared to the control group.

Specifically, subjective sleep quality in the intervention group decreased from 0.77 to 0.13, while it showed little change in the control group (0.06), and this difference was statistically significant (*P* = 0.03). Sleep latency scores also significantly decreased in the intervention group, from 1.52 to 0.88 (*p* = 0.02). Additionally, sleep duration improved in the intervention group (score decreased from 0.77 to 0.58), whereas it worsened in the control group (score increased from 0.86 to 1.08, *p* = 0.02). Habitual sleep efficiency scores decreased in the intervention group from 1.11 to 0.91, indicating improvement, while they increased in the control group from 1.38 to 1.55 (*p* = 0.003), suggesting a decline in sleep efficiency in the control group.

Furthermore, sleep disturbance scores decreased from 1.38 to 1.02 in the intervention group, whereas they increased to 1.44 in the control group (*P* = 0.004). In addition, daytime dysfunction improved notably in the intervention group (subscale score decreased from 1.47 to 0.77), whereas it increased in the control group (1.19 to 1.25, *p* = 0.003) (Table 4).

**Table 4:** Comparison of sleep quality scores between the two groups at two times

	Intervention Group (*n* = 36)	Control Group (*n* = 36)	Statistical Test Results	Sig
	Mean (SD)	Mean (SD)		
Subjective Sleep Quality				
At Intake	0.77(0.95)	0.47(0.94)	t-test: 1.36	n’s.
After 3 months	0.13(0.35)	0.41(0.89)	t-test: 2.14	0.03
Sleep Latency				
At Intake	1.52(0.65)	1.25(0.9)	t-test: 1.49	n’s.
After 3 months	0.88(0.46)	0.80(0.13)	t-test: 2.36	0.02
Sleep Duration				
At Intake	0.77(1.07)	0.86(93)	t-test: 0.35	n’s.
After 3 months	0.58(0.87)	1.08(1.02)	t-test: 2.22	0.02
Sleep Efficiency				
At Intake	1.11(0.94)	1.38(0.96)	t-test: 1.23	n’s.
After 3 months	0.91(0.84)	1.55(0.93)	t-test: 3.02	0.003
Sleep Disturbances				
At Intake	1.38(0.49)	1.33(0.47)	t-test: 0.48	n’s.
After 3 months	1.02(0.50)	1.44(0.65)	t-test: 3.03	0.004
Use of Sleeping Medication				
At Intake	0.16(0.56)	0.08(0.28)	t-test: 0.79	n’s.
After 3 months	0.08(0.50)	0.11(0.52)	t-test: 0.23	n’s.
Day time Dysfunction				
At Intake	1.47(0.69)	1.19(0.74)	t-test: 1.63	n’s.
After 3 months	0.77(0.48)	1.25(0.76)	t-test: 3.12	0.003
PSQI - Pittsburgh Sleep Quality Index				
At Intake	7.22(2.95)	6.58(2.97)	t-test: 0.91	n’s.
After 3 months	4.41(1.88)	7.11(4.14)	t-test: 4.51	0.000

Overall, the total PSQI score in the intervention group decreased from 7.22 to 4.41, indicating a substantial improvement in overall sleep quality (*p* < 0.001), whereas in the control group, the mean scores increased from 6.58 to 7.11, suggesting that sleep quality deteriorated in the control group compared with that in the intervention group. These findings underscore the positive and significant impact of the digital self-care intervention based on Iranian traditional medicine teachings on enhancing all dimensions of sleep quality in pregnant women.

Additionally, within-group changes assessed using paired t-tests revealed that the digital self-care intervention significantly improved various dimensions of sleep quality and maternal health literacy among pregnant women. The total PSQI score in the intervention group significantly decreased from a mean of 7.22 to 4.41 (t = 7.157, *p* < 0.001), signifying a marked improvement in the overall sleep quality. No significant changes were observed in the control group (t = 0.99, *p* = 0.32). The results for the other sleep quality subscales are presented in Table 5.

**Table 5:** The paired-samples t-test to investigate the difference between the participants’ pre-test and post-test scores in terms of sleep quality and maternal health literacy

Variable	Group	time	Mean	SD	T	*P*-Value
Subjective Sleep Quality	Intervention Group	At Intake	0.77	0.95	4.60	0.000
		After 3 months	0.13	0.35		
	Control Group	At Intake	0.47	0.94	0.29	0.76
		After 3 months	0.41	0.69		
Sleep Latency	Intervention Group	At Intake	1.52	0.65	6.46	0.000
		After 3 months	0.88	0.46		
	Control Group	At Intake	1.27	0.88	0.17	0.86
		After 3 months	1.25	0.80		
Sleep Duration	Intervention Group	At Intake	0.77	1.07	1.420	0.165
		After 3 months	0.58	0.87		
	Control Group	At Intake	0.86	0.93	1.13	0.26
		After 3 months	1.08	1.02		
Sleep Efficiency	Intervention Group	At Intake	1.11	0.94	1.070	0.292
		After 3 months	0.91	0.84		
	Control Group	At Intake	1.38	0.96	0.72	0.47
		After 3 months	1.55	0.93		
Sleep Disturbances	Intervention Group	At Intake	1.38	0.49	3.654	0.001
		After 3 months	1.02	0.50		
	Control Group	At Intake	1.33	0.47	0.84	0.40
		After 3 months	1.44	0.65		
Use of Sleeping Medication	Intervention Group	At Intake	0.16	0.56	1.784	0.083
		After 3 months	0.08	0.50		
	Control Group	At Intake	0.08	0.28	0.37	0.71
		After 3 months	0.11	0.52		
Daytime Dysfunction	Intervention Group	At Intake	1.47	0.69	5.562	0.000
		After 3 months	0.77	0.48		
	Control Group	At Intake	1.19	0.74	0.37	0.71
		After 3 months	1.25	0.76		
PSQI - Pittsburgh Sleep Quality Index	Intervention Group	At Intake	7.22	2.95	7.157	0.000
		After 3 months	4.41	1.88		
	Control Group	At Intake	6.58	2.97	0.99	0.32
		After 3 months	7.11	3.14		
Maternal Health Literacy Questionnaire	Intervention Group	At Intake	59.94	8.23	6.18	0.000
		After 3 months	68.16	4.76		
	Control Group	At Intake	61.38	6.12	2.33	0.02
		After 3 months	63.86	4.69		

Moreover, pregnancy-related health literacy among mothers in the intervention group significantly increased from a mean of 59.94 to 68.16 (t = 6.18, *p* < 0.001). In the control group, a modest and significant increase was observed from 61.38 to 63.86 (t = 2.33, *p* = 0.02), likely attributable to natural learning effects over time or exposure to the study setting.

These findings clearly demonstrate that the digital self-care intervention incorporating education on Iranian traditional medicine across six primary domains (eating and drinking, psyche, movement and rest, sleep and wakefulness, air and environment, retention of essential bodily substances and excretion of non-essential ones, with a focus on pregnancy-related changes) via a mobile health platform had a substantial impact on improving various dimensions of sleep quality and elevating pregnancy-related health literacy among pregnant women. In particular, the significant reduction in the global PSQI score in the intervention group (a decrease of 2.81 units) compared to that in the control group (an increase of 0.53 units) highlights the efficacy of this digital intervention in managing sleep disturbance during pregnancy.

## Discussion

This controlled quasi-experimental study demonstrated that a digital self-care intervention based on traditional Iranian medicine significantly reduced sleep disturbances in pregnant women. The intervention group showed a notable decrease in the Pittsburgh Sleep Quality Index (PSQI) score from 7.22 to 4.41, while the control group experienced a slight increase from 6.58 to 7.11, highlighting a significant between-group difference after the intervention. Improvements were observed in most PSQI subscales in the intervention group, aligning with enhanced prenatal sleep quality. Additionally, maternal health literacy increased significantly in the intervention group, from a mean of 59.94 to 68.16, highlighting a significant between-group difference post-intervention.

The underlying framework for improving sleep quality in pregnant women likely stems from the interactive components of the intervention, such as reminders and question-and-answer sessions, which enhance adherence and self-efficacy, as suggested in other digital health engagement models [30, 43]. The mechanism of action appears to involve multiple synergistic pathways. By integrating traditional medicine principles within an interactive mHealth platform, the intervention counteracts the cognitive and behavioral processes that perpetuate insomnia in pregnant women [31]. Psychological components, particularly mental health and emotional balance, probably reduce sleep-specific worry, rumination, and pre-sleep arousal while fostering non-judgmental awareness and acceptance, thereby mitigating daytime dysfunction and enhancing sleep efficiency [31]. Simultaneously, the marked increase in health literacy directly drives behavioral changes (e.g., improved nutrition, appropriate physical activity, and environmental adjustments), leading to physiological benefits, such as reduced systemic inflammation and better hormonal balance, which further supports restorative sleep [32]. A conceptual model, informed by structural equation modelling, confirmed that psychological factors exerted the strongest direct effect on sleep quality (path coefficient 0.658), whereas lifestyle factors mediated approximately 13.43% of the total effect. Culturally adapted content amplifies engagement and adherence, thereby strengthening these pathways [32]. This holistic, culturally congruent integration, reinforced by interactive elements, promotes adaptive responses over maladaptive sleep habits and distinguishes our intervention from generic telehealth approaches [31].

For instance, the psyche domain of Iranian medicine may target sleep disturbances and daytime dysfunction by addressing emotional well-being, whereas the movement and rest components improve sleep efficiency and duration. This suggests that psychological content improved mental well-being, while increased health literacy directly led to behavior change. Integrating the six domains of Iranian traditional medicine, with a focus on pregnancy-related changes, within the mHealth framework addresses cultural gaps in prenatal care.

By adapting these teachings to pregnancy changes through multimedia content (audio files, videos, pamphlets) and interactive elements (reminders, question-and-answer sessions via platforms such as Eitaa and Telegram), this digital educational intervention provides a comprehensive and accessible approach to pregnancy education. This intervention not only promotes self-care but also aligns with the cultural contexts of Iranian women and likely overcomes common barriers in prenatal education, such as limited access to low-resource areas. The absence of dropouts and high participant adherence highlight the program’s feasibility and position it as a model for addressing sleep disturbances during pregnancy through culturally adapted digital health interventions.

The slight increase in health literacy in the control group (2.51 units) may reflect natural progression through routine prenatal care or incidental exposure to health information, a common maturation effect in longitudinal studies, although the between-group differences in health literacy in our study confirm the intervention’s added value. The lack of change in the sleep medication subscale is attributed to low baseline use (exclusion of psychotropic users), which highlights the program’s non-pharmacological strength but limits insights into medication-dependent populations.

Comparing our findings with those of the existing literature highlights both thematic consistencies and differences regarding the effectiveness of mHealth interventions aimed at improving sleep quality. This includes traditional and integrative approaches as well as digital self-care methods. Regarding the impact of mHealth during pregnancy, our results align with those of Ameyaw et al. (2024) and Khademioore et al. (2023), who reported positive effects on maternal health. These studies indicate improvements in self-efficacy and physiological parameters, reflecting improvements in health literacy and sleep quality [7, 33]. However, the culturally specific features of our intervention distinguish it from more general platforms. As Asadollahi et al. (2025) noted, Persian-language pregnancy apps often lack depth and physician involvement, which may undermine their trustworthiness and effectiveness [19]. Similarly, Zamaninasab et al. (2023) and Asgari et al. (2023) highlighted the benefits of telehealth during COVID-19, such as reduced in-person visits and improved self-care behaviors, but reported varying effects of telehealth on specific outcomes, such as physical activity [4, 34]. In contrast, our quasi-experimental design in an urban Iranian sample provides strong within- and between-group evidence of improved sleep quality in the intervention group. Consistent with the present results, Khadem Husseini et al. explored the application of telehealth in maternal contexts with potentially higher generalizability and broader scope but with less cultural depth, and the findings indicated that, based on the Technology Acceptance Model (TAM), pregnant women exhibit good readiness and acceptance of remote prenatal care [35].

Regarding interventions for sleep quality, our results (a 2.81-unit PSQI reduction at the three-month follow-up) align with other studies, which also reported that digital cognitive-behavioral therapy for insomnia, with sustained post-intervention effects, is associated with more than twofold reductions in insomnia severity among pregnant women [36, 37]. Other studies also support non-pharmacological digital approaches, such as mindfulness and breathing exercises, for improving sleep disturbances in the perinatal period [38, 39], although the broader improvements across PSQI subscales in our study may stem from the multifaceted integration of Iranian medicine compared with their emphasis on psychological dimensions. Meneo et al. (2024), in a longitudinal review of digital psychological interventions, echo these results, noting moderate effects on sleep quality, but critique common issues in such studies, including small sample sizes and self-report biases, which are also present in our study [40].

Other studies have also emphasized the role of culturally adapted content in enhancing engagement and acceptance of traditional and integrative approaches [22, 23], which aligns with the outcomes of our study derived from incorporating Iranian traditional medicine teachings to increase adherence. This goes beyond the general digital tools reported in other studies, where cultural deficiencies hinder the usability of digital tools [20, 41]. Similarly, Lee et al. reported high user satisfaction with culturally aligned applications [42]. However, our study’s focus on the six lifestyle domains provides a more comprehensive framework, which may explain the superior improvements in health literacy compared with education-only interventions.

### Limitations

A primary limitation is the quasi-experimental design, which, while appropriate for this real-world context, introduces significant potential risks, such as selection bias, particularly through convenience sampling at the participant selection stage. This may severely limit the generalizability of the findings to broader populations beyond pregnant women in similar settings and further restricts the applicability of the results to non-representative samples. Additionally, while the data analyst was blinded, full blinding of participants and prenatal care providers was not possible given the interactive and educational components of the intervention, which is a common limitation in behavioral studies and may introduce performance or detection bias.

Furthermore, the highly restrictive inclusion criteria (e.g., no psychiatric disorders, no history of infertility or miscarriage, normal body mass index, and exclusion of those in rotating or night shifts) severely limited the generalizability of the findings to the broader population of pregnant women, particularly those with higher risk profiles or diverse socioeconomic backgrounds. The reliance on self-reported measures, such as the Pittsburgh Sleep Quality Index (PSQI) and the Maternal Health Literacy Questionnaire, introduces the potential for recall and social desirability bias, underscoring the need for more objective methods in future studies. Moreover, the lack of long-term follow-up beyond the three months limits our ability to assess the sustainability of the intervention’s effects. These limitations suggest that our findings should be viewed with caution and that future research should involve more diverse populations, longer follow-up periods, and more objective methods to enhance the validity of the results of the study.

## Conclusions

In conclusion, this study showed that a digital self-care intervention based on Iranian traditional medicine teachings can help improve sleep quality and pregnancy-related health literacy in pregnant women. The intervention group showed a notable reduction in the overall PSQI score (from 7.22 to 4.41) and improvements in most subscales, whereas the control group showed minimal change or even worsening. Maternal health literacy also increased significantly in the intervention group (from 59.94 to 68.16), highlighting the positive impact of this cultural digital approach on maternal health literacy. These findings align with the existing literature and suggest that integrating traditional elements with mobile technology could be a practical way to manage sleep disturbances during pregnancy, especially in cultural contexts such as that of Iran.

## Data Availability

The datasets generated and analyzed during the current study are not publicly available due to privacy restrictions of the participants but are available from the corresponding author upon reasonable requests.
